# Foreign

**Published:** 1841-03-27

**Authors:** 


### FOREIGN.

*Observations on Chorea.* By JOHN WEBSTER, M. D. Consulting Physician to the St. George's and St. James's Dispensary.—St. Vitus's dance was little known to the ancients, although Pliny and Galen are thought to have alluded to it; and it is still doubtful if the epidemic which prevailed so extensively in Germany in the 14th century was chorea, or the disease called Raphania. The most accurate description of St. Vitus's dance is that given by Sydenham and Dr. Hamilton. This complaint prevails among the poor, the badly fed and manufacturing town population of England; the higher and middle ranks being comparatively more free from its attacks. Girls are more subject to it than boys, and of 21 cases treated by Dr. Webster, 16 were in females, and but 5 in boys, or nearly 3 to 1.

Even when fatal, a much larger proportion of girls become victims to chorea than boys, as proved by the valuable report of the Registrar-General for 1840, which states that 20 females died of the disease, and only 4 boys, or 5 to 1, being the highest average in regard to the two sexes which the report contains. A very large number of the fatal cases occurred in the central and manufacturing districts of England, including the metropolis, and very few deaths in the more agricultural parts, whilst the northern counties, the pure agricultural and maritime districts, exhibited not a single fatal casualty, excepting one at Lancashire. The symptoms of chorea are so well known that no description of them was considered necessary by the author; and the treatment he found most successful consisted in active purging by aloes, jalep, and similar remedies, varying them according to circumstances; next the administration of tonics, such as Peruvian bark, ammonia, and camphor; and lastly the proper regulation of the ingesta, which was always found of the greatest importance, for if that point was neglected more purging was required, and the case thereby protracted. When the head appeared affected, leeches and blisters were useful; and if the uterine action also became implicated in the disease, then leeches and blisters to the loins, with the hip-path, and that added to the purgative remedies, were found to be of essential service. Of the 21 cases treated by Dr. Webster, only 1 proved to be fatal; but as in this instance the patient died of a fever which supervened to the St. Vitus's dance, and as the body besides was not examined, the author, from personal observation, could not bring forward any new fact regarding the etiology of chorea, although he expressed his opinion to coincide with those authors who consider the spinal cord and its membranes to be the primary seat of this singular disease.

At the conclusion of Dr. Webster's paper,

Dr. Wilks said that he had been led, by considerations founded on both physiology and pathology, to the conclusion, that chorea depended essentially on an affection of the cerebellum. In support of this, he alluded to the experiments of M. Magendie, which showed that certain injuries of the cerebellum, in animals, were followed by a loss of the directing power naturally possessed by the will over the voluntary muscles; and, in particular, to an experiment by that physiologist, which he had himself seen him accidentally perform, in which one of the crura cerebelli being divided the rabbit immediately lost all voluntary influence over its muscles, and began to rotate its body. He observed also that some cases of chorea which he had seen appeared highly favourable to this view. In one that had occurred at St. George's Hospital, a girl had been attacked with chorea soon after having fallen on the back of her head; all the usual remedies were employed without success; but the disease was ultimately removed by the repeated application of leeches and counter-irritation to the region of the cerebellum. Since that time Dr. W. had constantly treated chorea on the principles founded on the opinion he had been thus led to form, and with pretty constantly successful results.

Dr. Webster was aware that various authors had ascribed different parts of the nervous system, as the essential seats of the disease, marked by the convulsions of chorea. He regretted that he had not been able to perform any inspections of those who had died of the disease, and was, therefore, not in a position to form a decided opinion.

Dr. Addison having asked what Dr. Wilks considered to be the exact nature of the affection of the cerebellum, which gave rise to chorea, Dr. W. said, "it might be an affection marked by no palpable lesion; he believed, also, many different affections of the cerebellum might give rise to chorea, in the same manner as Dr. Abercrombie had shown that there were many different morbid conditions of the brain which were marked by the same symptoms; as for example in apoplexy, in which it was often observed that the same symptoms occurred in cases where blood was effused, in others where serum only was effused, and in others where no effusion at all had taken place. In the case to which he had particularly alluded, and which occurred after a fall, Dr. W. imagined that a condition of inflammation of the cerebellum must have existed.

Dr. Addison then said that the idea of the cerebellum being the essential seat of the disease in chorea seemed to him entirely disproved by the fact that in the cases which proved fatal that organ was not found appreciably altered, while in the cases where the cerebellum was actually diseased, no choreal symptoms occurred. He had never seen a case of fatal chorea in which the cerebellum appeared diseased; but he had seen many where from disease of the internal ear, or from other circumstances, acute inflammation had occurred in and about the cerebellum, leading to thickening of its membranes, effusion of albuminous matter, and even suppuration, and the formation of abscess—in which, in short, every circumstance that could produce irritation of the cerebellum had existed—and yet in none of these had any chorea occurred. He might apply the same observations with regard to the spinal cord, which was assumed to be the seat of the disease. He had had the misfortune to be able to examine several cases of chorea that had terminated fatally in his own and in his colleague's practice; but, after the most careful investigation, the only thing that was found remarkable was, the total absence of any appreciable lesion in the nervous centres. On the other hand, cases constantly occurred in which the spinal cord was importantly diseased, but in which no chorea existed; and he believed he might appeal to the experience of surgeons, that injuries of the spinal cord are seldom if ever followed by symptoms of this kind. He believed it would be found that in this disease, and all those related to it, which are marked by spasmodic action of the muscles, the seat of disease, whether such as produce appreciable lesion or not, was not so much in the medullary matter of the cord as in its membranes. So convinced was he of this, that it had become with him a matter of diagnosis, and, whenever he saw a case presenting, with other signs of disease within the vertebral column, convulsive action of the muscles, he at once considered the affection to be seated in the membranes of the cord, and not in its substance; whereas, if there existed paralysis of the muscles, he always conceived the nervous substance, and not the membranes, to be diseased. He thought attention had been given too exclusively to the nervous substance: to the neglect of the remarkable structure of the pia mater, and the other membranes which could not but exercise an important influence in the functions of the nervous system.

In confirmation of this opinion of Dr. Addison respecting the influence of diseases of the membranes, Mr. Davis related two cases, one of choreal affection, the other of epilepsy, both of which had occurred in consequence of local injury of the head, and both of which had been benefited only by the application of remedies to the part injured,—means which had produced complete cure in both.

Dr. Copland confirmed the author's statement as to the frequency of chorea among the poor of towns, by the fact that in the Manchester Infirmary there were seldom less than from twenty to thirty patients suffering from this disease. He wished to state his claims to the original idea of this affection being dependent on the spinal cord; as early as 1832 he had published his belief that it was the result of irritation, caused by disorder of the digestive system, and propagated through the ganglionic and vagus nerves to the spinal cord, from which it was again *reflected* to the nerves of the voluntary muscles. It was not till the following year that the same idea was brought before the Society by others.

The President said his observations had not led him to believe that chorea had any particular locality in the nervous system. In one case that had terminated fatally, he had found a cyst as large as a hazel-nut attached to the pineal gland; yet he should be far from thinking that that part was the seat of disease in chorea, though, in this case, its affection was, undoubtedly, the original cause of the symptoms. In another case, the cortical substance of the brain was in a peculiar state, and quite red by the turgidity of its vessels, and no other apparent disease existed; in a third, the chorea was consequent on a prick of the finger; yet it was not probable that the part evidently diseased in either of these cases, was the constant seat of the disease in chorea. He thought, also, it was erroneous to speak of the motions in chorea as involuntary; they seemed to him to be the results only of wrongly-directed will; the motions often commenced with such as were evidently voluntary, as picking the lips or fingers, and those gradually increased to real choreal movements. It was the same with hysterical affections of the muscles; in these cases the patient evidently willed the movements, but willed them at the wrong times, her mind not having sufficient control over her will to guide it aright; and so, in hysterical paralysis, the power of the muscles was not lost, but the will was not rightly exerted over them, and the patient was paralytic rather because she would not than because she could not move.

In illustration of the occasionally local origin of chorea, Dr. Kingston related a case, in which, after injury of the elbow in a young girl, a violent and long-continued, constant, involuntary motion, had existed in the arm.—*Lon. Med. Gaz.*—*Trans. Royal Med. & Chirur. Soc.*

We once made a careful examination of the of the body of a girl dead of chorea, and could discover no appreciable change whatever in any organ. It was a genuine case of death from the disease, wearing the child out by the continued fatigue and insomnia. The case occurred at the children's hospital of Paris: it was there found that any decided modification of the nervous system would cure most cases of the disorder, such as both cold and sulphur baths, tonics, or even purgatives.

---

*Cure of a Severe Gouty Pain by the Electric Ray.* By Dr. A. Chaeretes.—N. suffered three months without any evident cause from a very troublesome gouty pain in the first and second joints of the right fore-finger. He had used all the usual means against it, both internal and external, without benefit; on the contrary, the disease increased so much that he could not sleep during the greater part of the night, and in the day could not hold his pen. Thus he was going on one morning, trying to think of some new remedy by which he might possibly relieve his pain, and carefully avoiding meeting any one, lest an incautious touch might renew it now it was a little assuaged, when a fishing-boat drew near to sell the fish her men had just caught. Among them the patient remarked the electric ray, and being acquainted with its electric properties, he bought it and carried it home alive in a vessel filled with sea-water.—Although the fish was but small, yet it possessed a remarkable electric power; and the patient had scarcely touched it with the middle finger of the diseased hand, when he felt a severe shock and a peculiar pain in the elbow, with a numbness in the whole arm and of the fore-finger that had been the seat of the pain. After the lapse of a quarter of an hour, the numbness gradually ceased, and there remained only the peculiar sensation in the elbow, and that in a less degree. The fore-finger still gave indeed severe pain, but only when it was moved. Two hours after a second experiment was performed, of which the results were similar but more violent, and with this difference, that the fore-finger could soon afterwards be voluntarily moved without any pain, and when moved by the other hand, gave not the usual severe suffering, but only an unusual and peculiar sensation.—This condition continued without any material alteration till seven in the evening. At that time when the patient again repeated the experiment and touched the fish with the fore-finger itself, he felt a slight shock; but when he stimulated it with a piece of iron, he received seven shocks successively, and the fish soon after died.

That night the patient slept quietly; in the second he still felt only a numbness and weakness in the motions of the finger, which were completely removed by cold baths and cold douche in five days more. Since that time the finger has been quite freely used, and there has not remained the least trace of its former condition.

[Dr. Bouros, by whom this case is communicated, has added some notes respecting the uses which the ancients made of electric fish, but they are not of much interest, and may be found in the Dict. des Sciences Med. *art.* Poisson. He believes the fish used in this case was the Torpeo Narke. There is no reason given for calling it a gouty pain (*Gichtschmerze*) of the finger; was it not rather neuralgic?]—*Brit. and For. Med. Rev. from Casper's Wochenscrift.*

*Case of Convulsions of a peculiar kind* By G. Terrone.—R. S. a country labourer, of sanguine temperament, came to the hospital in March, 1838. He said he had always enjoyed good health, but had been constantly subject to poverty and hard work. Six months ago he began to suffer from a painful contraction of the back, which tormented him from time to time, and was not alleviated by any of the means he had employed. On examination, the affected part presented a curious appearance. The mass of muscles between the fourth and the eighth dorsal vertebræ, and for two inches width over the corresponding ribs rose up in the form of a compact, resisting, and hard globe; continued so for a few instants, and then disappeared and and left the part in its natural condition. Immediately after a similar swelling rose up on the opposite side; and so on, disappearing on one side when it appeared on the other. The diaphragm, and the abdominal and intercostal muscles participated in these convulsions, and they were accompanied by remarkable constriction of the throat, and frequently by slight hiccup. They occurred every five minutes, and when they ceased they left the patient in a perfect state of health.

The reader can form as good an opinion as the author does of the pathology of this affection. It is sufficient to add that it was gradually cured by cupping and the appiication of tartar-emetic ointment along the dorsal region of the spine, and by warm baths, with the occasional use of sulphuret anatomy and acetate of morphia.—*Ibid. from Annali Clinici dell' Ospedale degal' Incurabili.*

*On the Medical Action of the Bezoar of the Lacrymal Fossa of the Deer (Deer's tears) in Nervous Diseases.* By Dr. Löwenhardt.—The substance here described is a moist, strongly-smelling, fatty matter, which appears to the author to be secreted by the mucous membrane lining a fossa just below the anterior canthus of the red deer (*cervus elephas.*) It is found in this situation all the year, but in greatest quantity, it is said, during the rutting-season, it appears to serve by the diffusion of its powerful scent to attract or to keep off other kinds of animals. It exists only in the adult individuals of the species.

The smell of this bezoar is peculiar, very penetrating, strongly balsamic, much like that of amber mixed with a little benzoin, only still more pungent, and adhering half or all the day to the substances with which it comes in contact. Its taste is in like manner balsamic, and its most active principle is a peculiar resin which is of a yellow colour and lighter than water. Its analysis yielded resin (with a mixture of ethereal oil,) a very small quantity of fatty oil, wax, cellular substance, ammonia, and colouring matter, with muriate of soda and phosphate of lime; but it is probable that a part of these constituents are derived from the hair which is usually entangled in it.

It appears, by several cases which the author describes and refers to, to have a certain value in all the diseases in which castor, musk, valerian, and the other allied articles of the materia medica have been hitherto, and often unsuccessfully, employed; as hysteria, epilepsy, difficult menstruation, prolonged hooping-cough, &c.—The modes of administration which the author recommends are, 1st, in substance, in doses of from 5 to 10 or 15 grains, twice or three times, in pills with mucilage; and 2dly, in tinctures composed of one part of the bezoar to six parts of rectified spirit, or of ethereal spirit in doses of from ten, fifteen, or twenty drops twice or three times a day.—*Ibid. from Med. Zeitung.*

*On the Treatment of certain Diseases of the Brain.* By E. Copeman, Esq.

*Case* 1. Apoplexy occurring three times in the same individual.

In these attacks rapid recovery followed strong action in the alimentary canal, and the apoplexy was quite secondary to the gastric disturbance.

*Case 2. Hemiplegia cured without bleeding.*—Mrs. S., a farrier's wife, æt. 58, thin, but active and industrious, experienced a numbness in her left hand whilst preparing dinner for her family, which prevented her holding what she was using at the time. This occurred about noon, on the 23d of January, 1840, and was quickly followed by darting pains in the right temple. Feeling very unwell, she managed to get up stairs into her bed-room, and almost immediately lost her speech and the use of the left leg and arm. She had for some weeks previously felt occasional pain and numbness in the left forearm, which she considered to be rheumatism, and scarcely worthy of notice. I saw her at 3 P. M., and found her sitting in a large easy chair, with the following symptoms: left cheek partially paralyzed, and mouth drawn to the opposite side; articulation very imperfect; pain in right temple, but less severe than at first; left arm hanging by her side quite powerless; left leg imperfect in its movements, but not quite paralyzed; pulse feeble and indistinct; skin cool, with a tendency to shivering; nausea, and a sensation of faintness. Before I saw her she had swallowed with difficulty a little wine and water, and felt the better for it. The first question was, "Won't you bleed her as soon as possible?" I did not feel disposed to accede to this; but, from the depressed state of the system and cold surface, in preference administered a little more warm wine and water, and got her into a warm bed, with the head and shoulders a good deal raised.

Appl. Empl. Lyttæ Ampl. Nuchæ.

℞. Magn. Sulph. ʒvj.; Inf. Sennæ ℥vj.; Dec. Aloes c. ℥ij.; Tr. Jalap c. ʒij.; Tr. Hyos. ʒj.; Syr. Rhamni ℥ss. M. cochl. larg. iij. 2dis horis donec alv. bene respond.

11 *o'clock, P. M.*—Rather drowsy, but easily roused; breathing quite natural and easy; pulse 72, and soft; skin warm; speech less imperfect; left leg less powerless; pain in the forehead intermitting; says she feels more comfortable in every respect; blister drawing; bowels not yet relieved.

Cont. Mist.

24*th.*—7 *A. M.* Slept quietly several times for about an hour, without dreaming; had taken all the mixture, and swallowed the last dose without difficulty; skin warm; pulse 72; bowels acted powerfully three times; vomited once; can move the leg freely, and, to my astonishment, moves the forearm in the directions of flexion, extension, and rotation, to some considerable extent. I had intended to apply leeches to the right temple this morning, but the pain had so much abated that I postponed it for the present. Takes gruel, tea, and milk.

℞. Magn. Sulph. ℥ss.; Dec. Aloes c.; Inf. Sennæ *aa* ℥ij.; Tr. Hyos. ʒj.; Aquæ ℥ij. M. Cochl. larg. ij. 4tis horis.

9 *P. M.*—Much inclined to sleep, but quite sensible and collected when awake; pulse 60, regular, and soft; skin moist; can lift the left arm out of bed, and, when sitting up in bed, can raise her hand to the top of her head, but says the elbow feels heavy; urine scanty.

Cont. Mist.

25*th.*—Found her dressed and sitting up in an easy chair; can walk about the room, and move the arm almost without difficulty; no vertigo; pulse 72; bowels open; no drowsiness; very little distortion of the mouth.

26*th.*—Rather more pain in the temple, for which a blister was applied behind the ear.

Cont. Mist.

27*th.*—Blister drawn well, and pain in forehead removed; tongue milky; complains of slight numbness at the ends of the fingers, and feels languid.

℞. Inf. Gentian; Dec. Aloes c. *aa* ℥iv. M. Cochl. larg. ij. t. d. To take mutton broth with bread.

29*th.*—Improving; sits up several hours, and walks about the room.

Cont. Mist.

31*st.*—Complains of languor and faintness; tongue furred; pulse quiet.

℞. Quinæ Sulph. gr. viij.; Acid. Sulph. dil. ʒss.; Inf. Gentian c. ℥viiss.; Syr. Zingib. ℥ss. M. Cochl. larg. ij. t. d.

*Feb. 2d.*—Bowels not sufficiently relieved; ordered a wine-glassful of the first aloetic mixture every morning; tongue covered with yellowish fur, but has proper taste, and relishes food; can move the limbs freely; no distortion of the mouth. To remove the fur from the tongue and improve the secretion, to take a small dose of blue pill and rhubarb every night, and continue the quinine mixture.

14*th.*—Tongue clean.

Summat. Pil. noct. ter in hebdom. Cont. Quin.

Gets down stairs daily.

*May 4th.*—This patient has been gradually improving, and is now able to resume her duties as mistress of the family. The left arm is still weaker than the other, and will not bear prolonged exertion; but she can sew, &c. with considerable facility.

*August.*—Health good; takes no medicine but an occasional aperient.

Cases 3 and 4 are also illustrative of strong cerebral symptoms supervening after excesses in eating and drinking, and removed by very moderate bleeding, blistering, and purgatives. It is by no means uncommon to meet with cases of this kind; and the cerebral lesion is then very different from the hæmorrhage which occurs in true apoplexy; it is in fact merely a congestion of the brain, sometimes scarcely amounting to congestion, and rather to be classed with the nervous functional disorders of the organ, than natural vascular excitement.

From the case above narrated, and from others of a similar nature, I have been led to draw the following inferences:

1. That apoplectic and paralytic affections may take place in an extreme degree without organic disease of the brain.

2. That they often occur from other causes than pressure on the brain.

3. That bleeding, so far from being always necessary, is in many instances prejudicial.

4. That the effort of vomiting is not so prejudicial in these diseases as is generally supposed.

5. That counter-irritation, both external and internal, is a valuable means of affording relief to the symptoms immediately succeeding the attack.

1. *Apoplectic and paralytic affections may be severe without there being organic disease of the brain.*—In case 1, the first attack seemed to be the result of effusion and pressure on the brain: but it is clear that the second and third attacks, although much more severe at the commencement, could not have depended upon such a cause, or recovery would not have been so rapid and perfect. It is probable, therefore, that the first as well as the others arose from disorder of function, and not from organic disease. For the same reasons, the case of hemiplegia (No. 2) must have arisen from functional disturbance of the brain, and not from effusion of blood or serum.

"I have met with many instances in which it has been proved that not only a general derangement of the functions of the nervous symptoms producing apoplexy, but also partial effects of a similar nature, causing hemiplegia and paralysis, may take place, without any visible change of structure in the brain."—*Abernethy on Local Diseases.*

2. *These diseases often occur from other causes than compression of the brain.*—In the second attack, case 1, what are generally considered symptoms of compression were very marked; but had pressure been the cause of them, how could recovery have taken place so rapidly, and relief have been afforded immediately on the stomach rejecting its contents? This leads me to make a few remarks on the effects of pressure on the brain. I have often witnessed pressure on the brain from external violence without the symptoms attributed to compression. In the 18th volume of the Medical Gazette, a remarkable case is reported by Mr. Johnson, where a boy presented himself at the Norfolk and Norwich hospital with the thick part or screw of the breech pin of a gun firmly impacted in the frontal bone, the part which fastens on the stock projecting like a horn. The patient had no symptoms of compression till the pressure was removed by the extraction of the foreign body. In the same report are two other cases of fracture of the skull with depression to the extent of the thickness of the cranium, without symptoms of compression.

A little boy in my neighbourhood fell from a horse, receiving a severe wound in the forehead, and another at the back part of the head, where the cranium was fractured and depressed to the extent of the thickness of the bone. He was stunned by the blow at first, but soon recovered, without symptoms of compression, and without the depressed portion of bone being raised. He now enjoys good health, the pressure on the brain still remaining.

A child was affected with congenital hydrocephalus; the head gradually enlarged, until, at the age of 8 months, it had attained the following dimensions, viz:—Circumference, 24½ inches; from ear to ear over the top of the head, 15¼ inches; width between the frontal protuberances, 4¼ inches.

At the age of 13 months the size of the head was as follows:—Circumference, 26 inches; from ear to ear over the top of the head, 18 inches.

During the whole period referred to, this child had never been really ill. The appetite was good, and its senses unimpaired. Bowels were regular and healthy, and at the age of seven months had cut two teeth without difficulty. At the time of the last measurement of the head, I find the following report in my casebook:—"The child is much the same as to general health, its faculties are still tolerably perfect, its temper is cheerful, and it knows its attendants perfectly well. The body is more emaciated. About a week after this the child died suddenly with slight convulsions, but had not previously been worse than usual."

It is thus beyond a question that pressure upon the brain may exist to a greater extent than can be immagined to occur from loaded blood-vessels, without apoplectic or paralytic symptoms being produced. Compression is not the only cause capable of producing those symptoms, and I am inclined to believe that such a cause does not exist in a majority of the cases of apoplexy that present themselves.

3. *Bleeding, so far from being always necessary, is in many instances prejudicial.*—I am much inclined to the opinion that apoplectic and paralytic seizures are frequently diseases of debility, especially in persons advanced in years; and that the brain does not well bear detraction of blood in addition to the shock of the attack.

Celsus says, "Si omnia membra vehementa resolute sunt, sanguinis detractio vel occidit, vel liberat," and again, "Post sanguinis missionem, si non redit et motus et meus, nihil spei superest."

Hippocrates says that bleeding, except it relieves, kills. Boerhaave mentions a *cold phlegmatic* cause of apoplexy. Galen says "Paralyticorum parvus pulsus, languidus, tandusque est. Nonnullis rarus quoque, aliis creber, sed nonnihil inordinatè intermittens. Est enim parvus et tardus, *quia morbus est frigidus:* languidus, quòd *facultas infirmior.* Comitialium et attonitorum pulsus sunt similes." "Quorum trium morborum, frigidus, crassusque: aut omnino viscidus humor est causa."

In all cases of apoplexy or paralysis, in which the cause is dubious, "and such, on the first examination of them, the majority of them will probably be, it seems right to try the effect of correcting disorder of the digestive organs, with a view to alleviate nervous irritation, before we proceed to those severer methods, which the belief of the existence of organic or vascular disease in the brain would induce us to institute. For if blood-letting and counter-irritation be employed, in order to diminish vascular action; or if mercury be used to some extent, in order to induce the absorption of deposited substance; these measures must aggravate that disorder of the general health, upon which, in many instances, the nervous affection depends."—*Abernethy on Local Diseases.*

In the first attack, case 1, bleeding was carried to some extent, and the recovery was very tedious, a diminution in the functions of the nervous system remaining for a considerable time. In the 2d and 3d attacks, in which bleeding was not resorted to, the recovery was rapid and complete.

In the case of hemiplegia (case 2) the patient was several times in the course of her illness affected with symptoms indicating debility, which were relieved by tonics; and I am much inclined to think, if bleeding had been practised, the cure would have been more tedious and less perfect.

In case 5, in which bleeding was several times resorted to, the paralysis had become permanent, and frequently the general powers of the body have declined. The excitement caused by the heavy loss the patient sustained in the death of her husband, has produced amendment in her condition.

In case 4, in a young subject, bleeding was followed by coma, but whether as a consequence or not I cannot pretend to determine, as I did not see the state of the patient before he was bled. I merely record the fact. In case 3, the boy had recovered from the symptoms of disordered brain *before he was bled,* although probably according to the general opinion he was in circumstances especially demanding abstraction of blood.

4. *The effort of vomiting is not so injurious in these diseases as is generally supposed.* The second and third attacks in case 1, were very much benefited by it, and no prejudicial effect followed.

5. *Coumter-irritation, external and internal, is a valuable means of affording relief to the symptoms immediately succeeding the attack.* By *internal counter-irritation,* I mean irritation produced through the medium of the stomach and bowels, by emetics, purgatives, or nauseating medicines. I well remember, during my apprenticeship, being sent to visit a poor woman in Norwich, who had been taken in a fit. I found her apoplectic; and owing to a suspicion of her symptoms having been occasioned by taking laudanum, (which, however, was afterwards proved to have been unfounded,) a fellow pupil and myself proceeded to empty her stomach by means of the stomach-pump. The stimulus caused by the introduction of the tube, and injection of water into the stomach, roused her from her state of insensibility, and she quickly recovered. As a proof of the occasional good effects of external counter-irritation, in addition to what appears from the cases above recorded, I shall briefly refer to a circumstance which occurred at the Norfolk and Norwich hospital, during my residence in that institution. A man who had been the subject of general dropsy, and in a very weak condition, became insensible, and had all the symptoms of serous apoplexy. No bleeding could be practised on account of the debility present; neither, to the best of my recollection, could he be made to swallow any thing. The physician under whose care he was placed ordered blisters to be applied in succession, I might say, almost all over the body. On the back of the neck, on both arms, both thighs and legs, soles of the feet, and I think on the top of the head. In two or three days the patient regained his senses, and recovered from his apoplexy.

A question of great importance remains to be discussed, namely, whether there are symptoms generally present in cases of apoplexy and paralysis, which, by strict observation, may enable us to distinguish clearly between those which require bleeding, and those which would be better treated without it. I frankly confess my inability to answer it satisfactorily, and find that others have also failed in doing so. If I may be allowed to give an opinion, I should say that bleeding is unnecessary or prejudicial where the patient is sixty years of age or upwards; where the pulse is feeble, very frequent, intermitting, slow, or large, and inclined to double beat, (I have always found a pulse with double beat indicative of a state of system best relieved by diffusible stimuli,) where the respiration is laboured and accompanied with *cold* perspiration; where there is great mobility of the nervous system with weak muscles, whether the body be thin or *corpulent;* when the attack comes on soon after a full meal, or after great bodily or mental fatigue.

A quick, wiry, resisting pulse, flushed countenance, warm perspiration, noisy breathing, and a tendency to spasmodic muscular contraction, occurring in persons of an earlier age, seem to point out a necessity for resorting to abstraction of blood; but I believe there will be less danger in not bleeding in any case, than in always having recourse to it, where there are *some* circumstances indicative of the propriety of its employment.

I am fully aware that the observations that I have offered upon this most interesting subject are very imperfect, and, from my limited experience, cannot be conclusive either as regards the nature or the treatment of the diseases under consideration. My own mind, however, is strongly impressed with the truth of the inferences I have drawn, in opposition to the more prevailing opinions of the day. If they be correct, I shall be glad to find them confirmed by those who, from more extended experience, can form a better judgment; and if erroneous, I trust I may, from the same source, receive such instruction as may enable me to treat in the most effectual and best manner, those cases that may in future be submitted to my charge.

*Lon. Med. Gaz.*

---

*Therapeutic Effects of Platinum.*—The 48th number of the French Medical Gazette contains a long paper by Dr. Ferdinand Hoefer, on the therapeutic properties of platinum. Having made several preliminary experiments on animals, the Doctor commenced by taking the perchloride of platinum himself, and found that it was easily supported, in doses of one or two grains. On taking three grains, he experienced considerable headach, sensation of heat, and weight about the epigastric region, constriction of the throat, and inclination to vomit; but these symptoms, however, disappeared in half an hour. The perchloride of platinum was now administered to several patients affected with gonorrhœa and syphilis. The following cases illustrate the action of the remedy.

F. R., 46 years of age, had been frequently treated for syphilis, with mercury and sudorifics, which effected an apparent cure, but the symptoms soon returned, and the patient complained of sore throat; on examining which several syphilitic ulcers were detected on the back of the soft palate and neighbouring parts. He was ordered the perchloride of platinum, (four grains during the day.) On the twelfth day the ulcers were much improved in appearance, and on the twenty-third he was completely cured.

M. L., 27 years of age, had frequently contracted syphilis, of which he had been apparently cured by mercury; but about two months after the last course, he experienced considerable pain in the bones at night, and a syphilitic eruption broke out on the upper part of the right thigh. Dupuytren's pills and Barège baths were employed, without avail. He was now ordered to take the following. Perchloride of platinum, ten grains; extract of guaiacum, eighty grains; powdered liquorice, enough to make twenty pills. Of these four were given during the day; and the patient was quite well in a fortnight.

F. J., 30 years of age, had the face, limbs, and chest covered with a syphilitic eruption. Sulphureous baths, mercury, iodine, &c., were tried, without success. He took a mixture, containing five grains of the perchloride of platinum in the day, and washed the eruption with a lotion, composed of one scruple of the same remedy to twenty-five of water. A cure was obtained on the fifteenth day.

The following are the conclusions at which Dr. Hoefer has arrived, from his experiments.

1. The perchloride of platinum is poisonous, at the dose of twenty grains; the bichloride of platinum and iodine, at the dose of forty grains; but they are less poisonous than chloride of gold or corrosive sublimate.

2. A concentrated solution of the perchloride, when applied to the skin, produces smart itching, followed by a slight cutaneous eruption of the part.

3. The bichloride of platinum and iodine does not produce any local irritation of the skin. The perchloride is an excellent remedy in the treatment of inveterate secondary syphilis; the bichloride is more applicable to recent cases and to rheumatism.

4. Platinum then must be ranked in the class of alterants, by the side of gold, mercury, iodine, and arsenic. It does not produce any of the dangerous effects which have been attributed to mercury, and differs from it in its mode of action.—*London Provincial Medical and Surgical Journal.*

---

*Ioduret of Iron.*—When a solution of the ioduret of iron comes in contact with atmospheric air, a portion of the iron is oxydized, and a corresponding quantity of the iodine is precipitated. Water saturated with sugar possesses the property of preventing the oxydization of the iron; hence the remedy should always be employed in the following manner. Take of simple syrup, 200 scruples; liquid ioduret of iron, one scruple; mix. A teaspoonful of this mixture contains a grain of the dry ioduret.—*Ibid., from Journal de Chimie.*

---

*Novel Lawsuit.*—A most singular cause is about to be tried before one of the French law courts. When the celebrated Pinel died in 1826, his pupils MM. Esquirol, Alibert Recamier, Rostan, &c., thought it right to examine his body, and M. Esquirol prepared the defunct professor's skull with the greatest care, and preserved it as a *souvenir* of his master. But on the death of M. Esquirol, M. Scipio Pinel comes forward, and is about to institute a lawsuit for the recovery of his father's skull, asserting that he is legally entitled to it.

